# First experiences of a hospital-based 3D printing facility – an analytical observational study

**DOI:** 10.1186/s12913-023-10511-w

**Published:** 2024-01-04

**Authors:** Christian Kveller, Anders M. Jakobsen, Nicoline H. Larsen, Joakim L. Lindhardt, Thomas Baad-Hansen

**Affiliations:** 1 Department of Orthopedic Surgery, Aarhus University Hospital, Palle Juul-Jensens Boulevard 99, 8200 Aarhus, Denmark; 2 Department of Plastic and Breast Surgery, 3D Innovation, Aarhus University Hospital, Aarhus, Denmark; 3 Department of Dentistry, Section for Oral and Maxillofacial Surgery, Aarhus University, Aarhus, Denmark

**Keywords:** 3D printing, 3D-printed, Patient-specific surgical instruments, Patient-specific anatomical models, Lead time

## Abstract

**Purpose:**

To identify the clinical impact and potential benefits of in-house 3D-printed objects through a questionnaire, focusing on three principal areas: patient education; interdisciplinary cooperation; preoperative planning and perioperative execution.

**Materials and methods:**

Questionnaires were sent from January 2021 to August 2022. Participants were directed to rate on a scale from 1 to 10.

**Results:**

The response rate was 43%. The results of the rated questions are averages. 84% reported using 3D-printed objects in informing the patient about their condition/procedure. Clinician-reported improvement in patient understanding of their procedure/disease was 8.1. The importance of in-house placement was rated 9.2. 96% reported using the 3D model to confer with colleagues. Delay in treatment due to 3D printing lead-time was 1.8. The degree with which preoperative planning was altered was 6.9. The improvement in clinician perceived preoperative confidence was 8.3. The degree with which the scope of the procedure was affected, in regard to invasiveness, was 5.6, wherein a score of 5 is taken to mean unchanged. Reduction in surgical duration was rated 5.7.

**Conclusion:**

Clinicians report the utilization of 3D printing in surgical specialties improves procedures pre- and intraoperatively, has a potential for increasing patient engagement and insight, and in-house location of a 3D printing center results in improved interdisciplinary cooperation and allows broader access with only minimal delay in treatment due to lead-time.

**Supplementary Information:**

The online version contains supplementary material available at 10.1186/s12913-023-10511-w.

## Introduction

3D printing has gained increasing attention as the technology behind has improved, particularly in orthopedic and maxillofacial surgery, and it is likely to play a significant role in healthcare and clinical practice. This is due to the ability to create patient-specific anatomical models (PAM) and patient-specific surgical instruments (PSI) [1–3], which can customize devices and procedures to the patient, reducing the duration of surgical procedures, treatment, and recovery while improving accuracy and outcomes. Numerous medical specialties are already using 3D printing to manufacture custom surgical guides, implants, orthotics, and anatomical models for preoperative planning or education [4, 5].

In addition to preoperative planning, PAM’s have the potential to enhance patient education concerning their condition and facilitate informed shared decision-making concerning treatment options available for their specific affliction, and the benefits and/or risks involved in treating it [6, 7].

However, many surgeons still have to rely on external providers for their 3D printing needs [8]. Worldwide, the revenue of the 3D printing industry exceeds $4 billion and is fast growing, with 13.1% of revenue attributed to the medical sector [9]. Establishing a hospital-based specialized 3D printing provider, enables faster and wider use in different specialties and reduces patient risk by removing the need to transfer personal and medical information to a third party [10].

In 2018, a 3D printing center was established at our facility, capable of printing both personalized anatomical models as well as cutting guides based on CT scans. This project attempts to discern the subjective value, if any, in-house 3D printing brought to the clinicians who utilized it for individual patient treatments.

## Aim

The aim of the present study was to identify the clinical impact and potential benefits of in-house 3D-printed PAM and PSI through a questionnaire, focusing on three principal areas: (1) patient education; (2); interdisciplinary work and communication (3) preoperative planning and perioperative execution.

## Materials and methods

### Study design and population

This study was conducted as an Institutional Review Board approved survey. A questionnaire was sent to all clinicians who had previously ordered a patient-related 3D-printed PAM or PSI at Aarhus University Hospital 3D Printing Center from January 2021 to August 2022. The 3D-printed object was linked to a personal identification number and the questionnaire was sent shortly after surgery or finalized treatment. Respondents were given 2 months to answer. Reminders were sent after 2 weeks to non-responders.

Questionnaires were distributed using SurveyXact software, a web-based software platform designed to support data capture for research studies. By completing the questionnaire, the respondents acknowledged that they were giving their consent to participate in the study. All responses were received anonymously. Clinicians could receive multiple questionnaires for different patients. No participants were financially compensated for completion of the survey.

### Questionnaire

The questionnaire, developed for this study in Danish, included 14 questions regarding information/education of patients undergoing surgery, questions concerning preoperative planning and perioperative execution, and communication with the in-house 3D printing center. The answers could be rated on a scale from 1 to 10, with 1 being ‘none’ and 10 being the highest value. One question regarding the scope of procedure asked the surveyed to rate from 1–10–1 being ‘less invasive’ and 10 being ‘more invasive’. The participants could add supplemental comments to certain questions. Data obtained from the web-based software platform was exported to Microsoft Excel. Descriptive data was expressed as percentage. An English version of the questionnaire is included in the supplementary material.

**Fig. 1:**
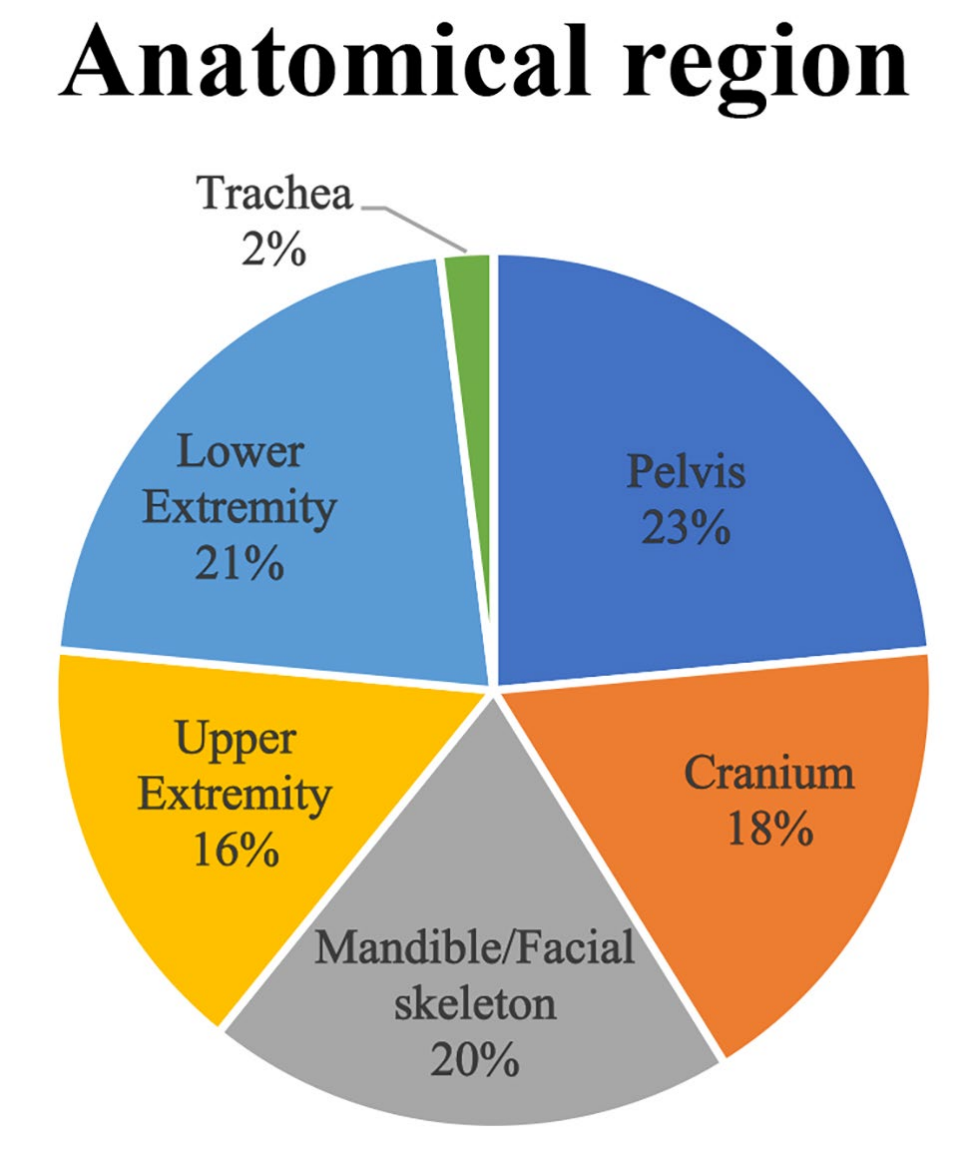
The anatomical region either printed or for which the 3D-printed tool was intended

A total of 118 sets of 3D-printed PAM or PSIs were ordered between January 2021 to August 2022. 51 responses were received; the response rate was 43%. A majority of the surveyed were affiliated with the Orthopaedic (61%) or Maxillofacial Surgery Departments (21%), and only to a limited extent the departments of Otolaryngology (4%), Plastic Surgery (4%) and Neurosurgery (2%). 80% of the surveyed were senior specialists within a group consisting solely of specialists. Anatomical regions of interest printed varied (Fig. 1). The results of the rated questions are given as averages.

**Fig. 2:**
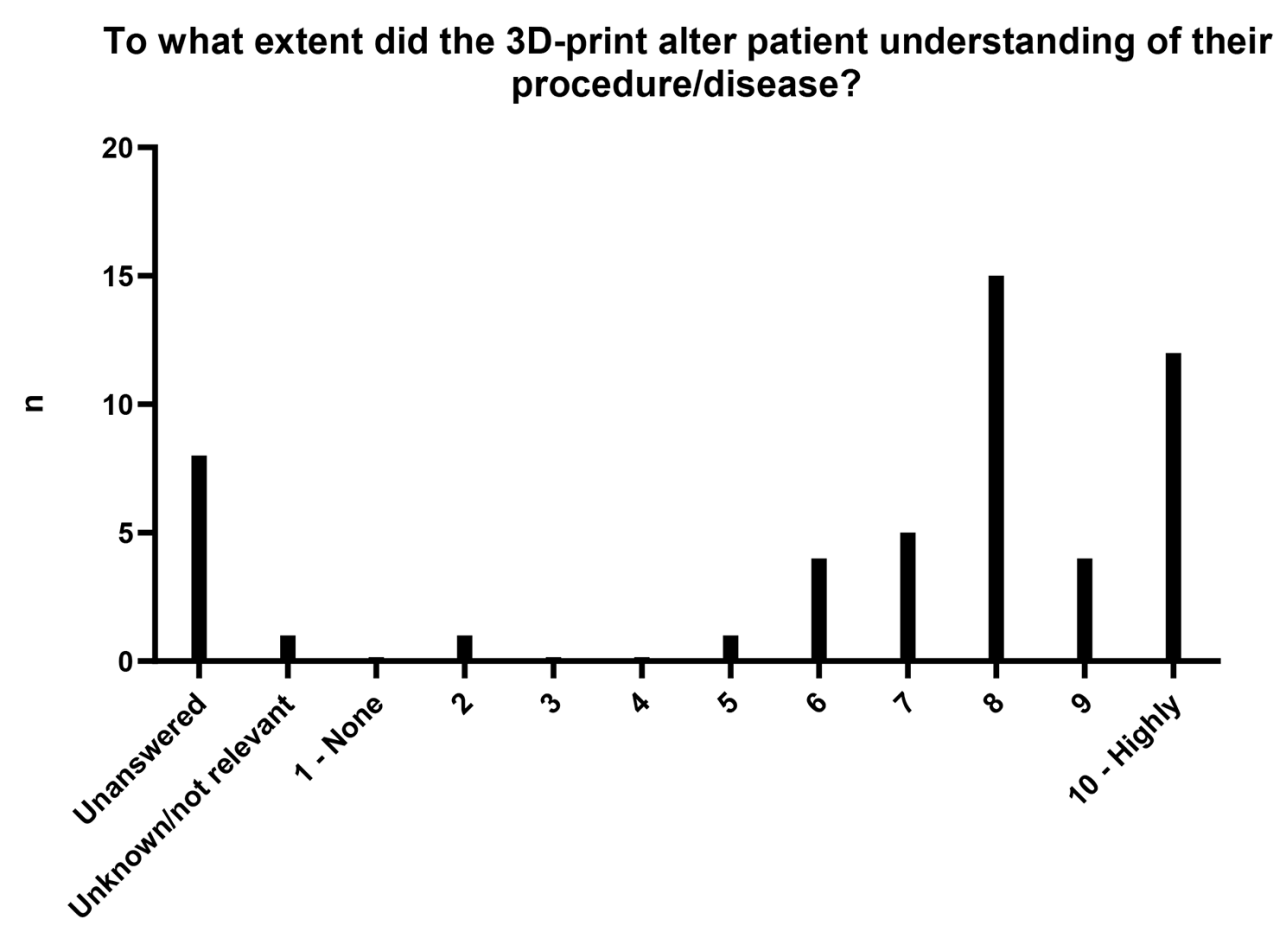
Clinician-reported estimation of the degree the PAM/PSI altered patient’s understanding of their procedure/disease

84% of clinicians reported using 3D-printed objects in informing the patient about their condition and/or expected procedure. Clinician-reported improvement in patient understanding of their procedure/disease was 8.1 (Fig. 2).

The importance of in-house placement and its derivatives were rated 9.2. 96% of responders reported using the 3D model to confer with colleagues. An increase in interdisciplinary cooperation as a result of the 3D-printed object was reported at 8.5. Reported delay in treatment due to 3D printing lead-time was 1.8.

**Fig. 3:**
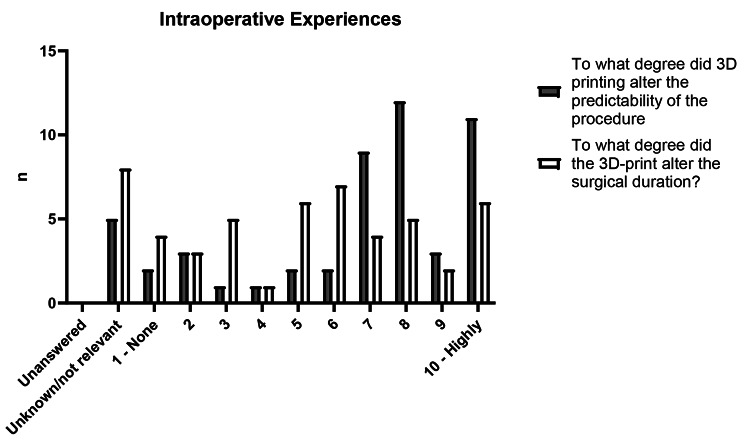
Intraoperative clinician experiences

**Fig. 4:**
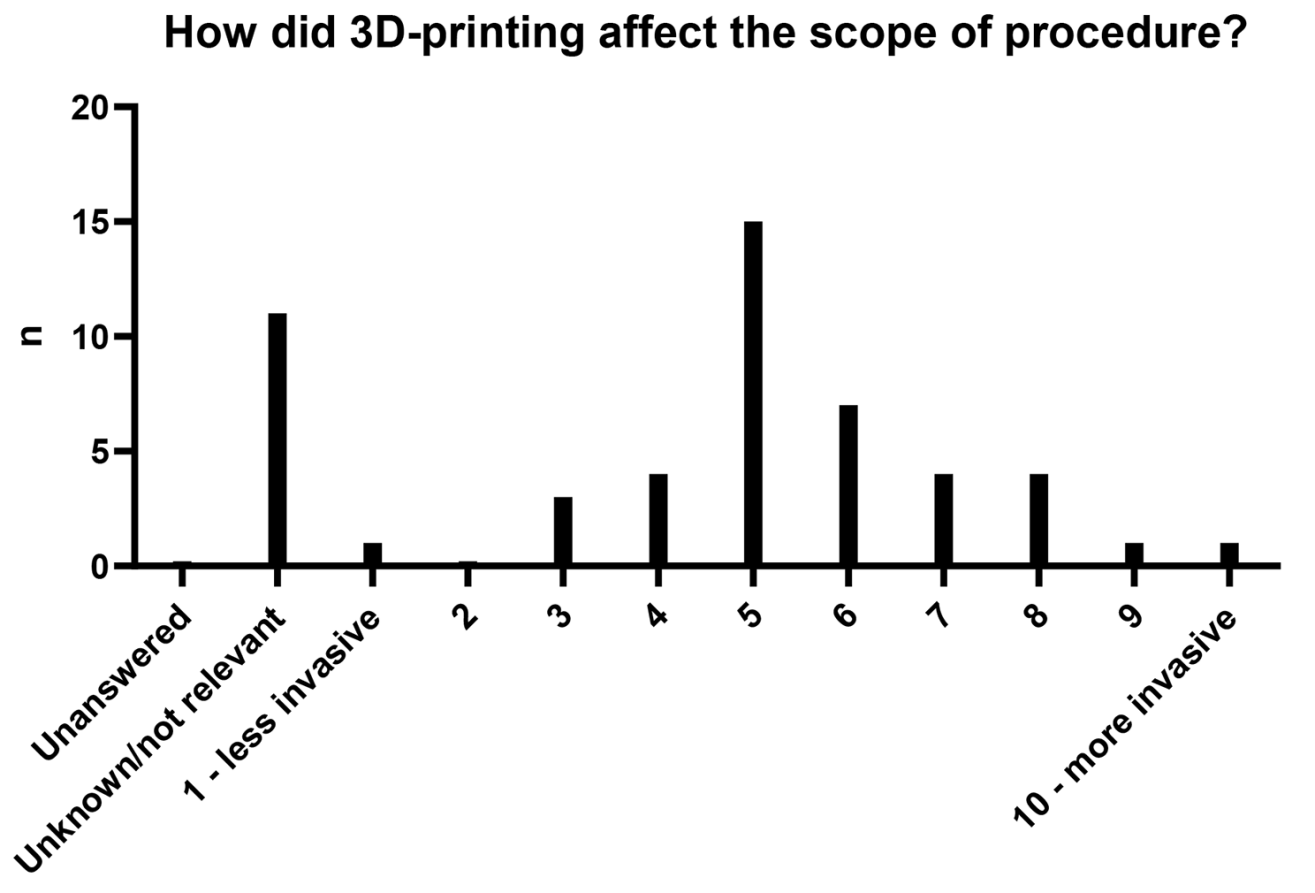
Reported change in invasiveness of procedure as a result of the 3D-printed object

The reported extent to which 3D printing was found to have impacted preoperative planning was 6.9. The improvement in the perceived preoperative confidence ahead of the procedure was 8.3. The alteration in intraoperative predictability was reported 7.2 (Fig. 3). The degree with which the 3D-printed object affected the scope of the procedure, in regard to invasiveness, was 5.6, wherein a score of 5 is taken to mean unchanged in this specific question (Fig. 4). Reduction in surgical duration was rated 5.7 (Fig. 3).

Finally, a total of 18 free text comments were written. 1 of 18 were deemed negative. This respondent commented on the lack of rigidity of a 3D-printed cutting guide (a PSI) which deformed during surgery – making the cutting more difficult. The remaining comments were deemed either positive or neutral pertaining to the specific case and/or 3D-printed object. Two commented that the PAM allowed for pre-operative preparations allowing the surgeons to utilize pre-adapted standard implants rather than expensive custom-made implants. Another two commented that the procedure was cancelled as a result of the PAM showing the previously planned procedure unfeasible.

## Discussion

Over the last decade 3D printing has become an increasingly important technology in the surgical field. Earlier surgical studies have primarily had aims based on the possible surgical advantages, while only few studies have focused on patient education and shared decision-making. Suboptimal health literacy has been shown to be an independent risk factor for poor health outcomes, especially in cancer patients [11]. For the patient, PAMs opens an avenue to more easily facilitate increased understanding of their condition, as well as solidifying the foundations on which the clinician can base shared decision making with the patient upon [6, 7, 12, 13]. Our results show that, although the clinicians are not obligated to use the PAM/PSI in informing the patient, the majority did and, in the clinicians assessment, it increased patient understanding of their condition or upcoming procedure. A recent scoping review on the use of PAMs in patient communication found almost universal positive results, but also that patient communication was often conflated with patient education in these studies despite being different, albeit overlapping, entities [14]. Pugliese et al. [15] found that, in adolescents, while feelings towards the PAMs were generally positive and improved rapport, a non-negligible minority felt more anxious about their condition, when confronted with their model. Van de Belt et al. [6] found that while PAMs increased patient understanding, that increased understanding also had emotional repercussions as the patients were confronted with their conditions.

For surgical navigation the tactile feedback of 3D-printed anatomical models significantly aid in comprehension compared with virtual 3D reconstructions or 2D imaging [16, 17]. The advantage conveyed by 3D printing for preoperative planning, surgical navigation and reduced surgical duration is weighed up against the disadvantage of increased lead-time, barrier-to-entry and additional costs in hardware and software necessary [3, 18]. Another disadvantage of 3D-printed anatomical objects is a result of the mechanical properties of the material – with a rigidity unable to reproduce the compliance of biological tissues or a fragility unable to withstand the sterilization process and thus safely handled during surgery or physical deformation during surgery, with thinner structures being more susceptible to deformation. The total cost of a PAM/PSI has been reported in the range of 150–700€ per patient [3]. The additional cost, however, may be offset by the reduction in operative duration as a consequence of the PAM/PSI being used in pre-operative planning – time spent outside and inside the operating being unequal in terms of expenses [19].

Our study, which mainly involved specialists in various fields, found that the use of 3D-printed models and surgical tools increased clinician preprocedural confidence, intraoperative predictability, and reduced procedural duration when performing PAM/PSI-assisted surgery. This demonstrates the potential value of 3D printing, not just as a guiding tool for the inexperienced, but to hone difficult or rare procedures, likely improving results, and subsequent patient outcomes, of even the thoroughly experienced specialist.

Our study focused on the advantages of 3D printing within an in-house production unit but does not directly compare the in-house lead-time with that of outside commercial manufacturers. In-house 3D printing allows for the use of 3D printing even in the acute setting, allows for immediate reconstruction following excision of malignancies [20, 21], and may create new avenues in surgical techniques and clinical applications that were once hamstrung by the delay of outsourced 3D-printed objects [22–24]. Furthermore there may also be a cost-mitigating factor surrounding in-house 3D printing when taking into account the potential cost-saving measures PAM’s can provide in time-saving measures outside a costly operating room and allowing the use of prepared or pre-bended standard implants compared to otherwise using expensive custom-made implants [25].

Currently, our in-house 3D printing center produces surgical guides, anatomical models used in clinical practice and experimental implants for use in animal studies. Ascertaining the true value of 3D printing in the clinical setting is difficult as the technology is often used in cases preoperatively suspected of being challenging and a high degree of selection bias would thus be expected in regard to outcomes. Conversely, if the technology was used widely for routine cases the benefits might be obscured, especially if routine cases vastly outnumber challenging cases.

## Conclusion

In conclusion, our study found that in-house 3D printing of PSI/PAMs has the potential to improve surgical procedures and increase patient engagement and insight and reflects the first experiences with in-house 3D printing of a single institution. The study was conducted at a major university hospital, delivering highly specialized treatment.

To clinicians, the utilization of 3D printing in surgical specialties improves procedures pre- and intraoperatively while the scopes of surgery are largely unaffected, has a potential for increasing patient engagement and insight, and in-house location of a 3D printing center results in improved clinical cooperation and allows broader access, with only minimal delay in treatment due to 3D printing lead-time.

### Electronic supplementary material

Below is the link to the electronic supplementary material.

**Supplementary Material 1:** English 3D printing Questionnaire

## Data Availability

The raw extracted data contains personal identification information, and an anonymized version can be made available upon request to the first author.
