# Supplementary material for: First experiences of a hospital-based 3D printing facility – an analytical observational study
Source: BMC Health Serv Res. 2024 Jan 4;24:28. doi: 10.1186/s12913-023-10511-w (PMC10768152; doi:10.1186/s12913-023-10511-w)
Supplement: Supplementary file 1 — Supplementary Material 1: English 3D printing Questionnaire [file 12913_2023_10511_MOESM1_ESM.docx]

English 3D printing Questionnaire:

1. Job Title

2. Department

3. Anatomical region

4. The 3D model was used in informing the patient?

5. To what extent did the 3D print alter patient understanding of their procedure/disease?

6. To what extent did the in-house 3D printing center affect your work compared with commercially available 3D printing firms in terms of communication, design, and lead time?

7. The 3D-model was used to confer with colleagues?

8. To what extent did the 3D print increase interdisciplinary cooperation?

9. To what degree was treatment delayed due to in-house 3D-printing lead-time?

10. To what degree did 3D printing affect preoperative planning?

11. To what degree did 3D printing improve perceived preoperative confidence with regard to surgical procedure?

12. To what degree did 3D printing alter the predictability of the procedure?

13. How did 3D printing affect the scope of procedure?

14. To what degree, if any, did 3D printing reduce surgical duration?
